# Can heat stress affect the psychophysiological responses and locomotor demands of young soccer players during small-sided soccer games?

**DOI:** 10.1371/journal.pone.0351116

**Published:** 2026-06-09

**Authors:** Ersan Arslan, Yusuf Soylu, Ana Filipa Silva, Osman Yilmaz, Bulent Kilit, Neslihan Akçay, Lukasz Radziminski, Ladislav Batalik

**Affiliations:** 1 Faculty of Sport Sciences, Tokat Gaziosmanpasa University, Tokat, Türkiye; 2 Escola Superior Desporto e Lazer, Instituto Politécnico de Viana do Castelo, Rua Escola Industrial e Comercial de Nun’Álvares, Viana do Castelo, Portugal; 3 Research Center in Sports Performance, Recreation, Innovation and Technology (SPRINT), Melgaço, Portugal; 4 School of Physical Education and Sports, Osmaniye Korkut Ata University, Osmaniye, Türkiye; 5 Hasan Dogan Faculty of Sports Sciences, Karabuk University, Karabuk, Türkiye; 6 Department of Physiology and Biochemistry, Gdansk University of Physical Education and Sport, Gdansk, Poland; 7 Department of Physiotherapy and Rehabilitation, Faculty of Medicine, Masaryk University, Brno, Czech Republic; 8 Department of Rehabilitation, University Hospital Brno, Brno, Czech Republic; 9 Department of Public Health, Faculty of Medicine, Masaryk University, Brno, Czech Republic; 10 Rehabilitation Clinic, Faculty of Medicine, Masaryk University, Brno, Czech Republic; Portugal Football School, Portuguese Football Federation, PORTUGAL

## Abstract

The study compared the effects of three different heat stress conditions on the psycho-physiological responses and locomotor demands of young players in different small-sided soccer games (SSGs). Sixteen soccer players (age: 16.5 ± 0.5 years) performed 2-a-side and 4-a-side SSGs under three environmental heat stress conditions: low environmental heat (LEH) ≤ 23.9°C, moderate environmental heat (MEH) 24.0–27.9°C, and high environmental heat (HEH) 28.0–32.9°C. Players’ heart rate (HR) responses and total distance covered (TDC) were continuously monitored for all SSGs; the rating of perceived exertion (RPE) and visual analog scale (VAS) were used after each bout. Tympanic temperature (TT) was recorded daily before and after the SSGs. The results demonstrated that for both 2-a-side and 4-a-side SSGs, significant main effects of temperature were observed for HR, %HR_max_, RPE, and VAS responses (all p < 0.05), indicating progressively increased cardiovascular strain and perceptual load under higher heat stress conditions. In contrast, no significant main effects of temperature were found for TDC and TT responses in either game format. Interaction analyses revealed significant temperature × bout effects for most psychophysiological variables in both SSG formats, including HR, %HR_max_, RPE, and VAS responses (all p < 0.05), suggesting that responses to heat stress varied across repeated bouts. However, no significant interaction effects were observed for TDC or TT in the 4-a-side SSGs, while only limited interaction effects emerged for TDC in the 2-a-side SSGs format. Overall, the findings indicate that heat stress substantially amplifies cardiovascular and perceptual responses during SSGs, with effects modulated by game format and bout structure. In contrast, TDC and TT appear less sensitive to these conditions. Coaches may use this evidence to manage players’ internal and external load and optimize their team’s performance across various heat-stress conditions.

## Introduction

Environmental conditions (e.g., high altitude, cold, heat, and humidity) play an essential role in modulating soccer players’ performance, which is a reason for concern for entities such as the FIFA Medical Committee and coaches, physicians, and medical staff [1]. Numerous teams worldwide often must compete under challenging weather conditions. Therefore, the effects of environmental conditions (e.g., heat stress) on soccer players’ performance have recently become a popular area of attention in sports science [2,3]. Physical activity during heat is associated with several physiological responses that can affect fatigue development [4]. During prolonged exercise, increased temperature changes the cardiovascular, respiratory, and central nervous systems, as well as muscular factors [5]. Disturbances in stroke volume and cardiac output, accompanied by elevations in skin and blood temperature, as well as simultaneous interference with fluid-electrolyte balance, limit oxygen delivery to exercising muscles and reduce the efficiency of aerobic metabolism in adenosine triphosphate resynthesis [5–8]. Consequently, changes in the rate of energy system contribution, especially anaerobic glycolysis and creatine phosphate hydrolysis, can negatively affect exercise ability [9,10]. Furthermore, elevated wash-out of carbon dioxide results in increased ventilation during submaximal exercise in heat [5].

All these alterations could significantly affect internal and external responses in exercising athletes. As mentioned above, disturbances in stroke volume are partially compensated by increased submaximal heart rate (HR) values in hot environments [5]. Abellán-Aynés et al. [11] confirmed the impact of hot conditions on the cardiovascular system by demonstrating reduced HR variability, which may result from lower parasympathetic activity [12]. Recently, Kang et al. [13] reported that significantly higher HR responses accompanied by increased ratings of perceived exertion (RPE) occur during small-sided soccer games in young players at higher temperatures. RPE disturbance can be determined by several psychophysiological factors such as thermal discomfort [14], elevated core body temperature, and increased physiological load [15].

Several authors have previously investigated the effects of heat stress on the physical performance of soccer players. For example, the total distance covered (TDC) by midfielders during a match played at temperatures above 21°C was significantly lower (4%) than in colder conditions [16]. This was further supported by Mohr et al. [17], who demonstrated that physical performance during soccer matches played under hot conditions (31°C) markedly deteriorated in the final 15 min of the game. Furthermore, a comparison of physical match performance at temperatures of 21°C and very hot conditions (43°C) revealed a significant drop in TDC and high-speed running (HSR) (7% and 26%, respectively) [6]. Draper et al. [18] reported in their review that although the detrimental effect of hot temperature on TDC and HSR was observed in several studies, other researchers found no significant differences in these physical performance components. Thus, further investigations on the effects of heat stress on external responses in soccer are warranted.

Small-sided games (SSGs) are among the most popular training drills that effectively replicate soccer matches [19]. These game-based exercises provide an effective conditioning stimulus and allow them to develop technical-tactical skills [20]. Several factors affect players’ behavior and the intensity of the game during SSGs. Variables such as the number of players, pitch size, coach encouragement, game rules, and work-rest ratio were identified as significant [21–23]. Among others, the number of players participating in the game is a critical determinant of player behavior, ultimately resulting in variations in psychophysiological and locomotor responses [24,25]. For example, 2-a-side and 4-a-side SSGs are typically characterized by HR responses above 85% of the maximum HR (HR_max_), constituting a considerable cardiovascular stimulus for players [26]. Although acute responses to SSGs format manipulation have been previously characterized, to the best of our knowledge, no studies have investigated the effects of heat stress on variations in psychophysiological and locomotor responses in these SSGs. Research on the effects of various conditions on players’ psychophysiological and locomotor responses during SSGs can help coaches understand their impact on performance and ultimately adjust training interventions to preserve player performance. As no study in this regard has been conducted so far, and given its relevance, the purpose of this study was to measure the psychophysiological and locomotor responses to two SSG formats (2-a-side and 4-a-side) under three different heat stress conditions. In particular, we tested two sub-objectives: (i) the influence of heat stress on the main outcomes and (ii) the influence of heat stress on the performance of repeated bouts of SSGs.

## Methods

### Experimental approach to the problem

A repeated-measures design was used to compare the effects of heat stress on the psychophysiological responses and locomotor demands of the 2-a-side and 4-a-side SSGs. These types of game-based drills, called extreme and SSGs, have been shown to reflect similar demands for high-intensity training [24]. In accordance with the purpose of this research, the measurements were completed over 13 days under different heat-stress conditions during the pre-season training period (from mid-August to the end of August). Due to environmental constraints, heat stress conditions were applied in a fixed sequence. To minimize potential sequence effects and heat stress-related physical fatigue in players, a short-term research design was employed. Furthermore, players were provided with at least 48 hours of passive rest between sessions; during this time, no additional training or strenuous physical activity was performed. All SSGs were performed as part of regular training sessions after a standardized 10-minute warm-up section consisting of low-intensity running and dynamic stretching with short-distance passing. Young soccer players were continuously monitored using a GPS-enabled watch HR monitor (Polar M430, Polar Electro Oy, Kempele, Finland) during the SSGs [27]. When relative humidity was at or below 50%, the risk of heat injury was defined as “low” if WBGT was below 24°C, “moderate” between 24 and 28°C, and “high” between 29 and 33°C. The environmental conditions, including temperature and relative humidity, were defined as low environmental heat stress (LEH) ≤ 23.9°C, moderate environmental heat stress (MEH) 24.0–27.9°C, and high environmental heat stress (HEH) 28.0–32.9°C as classified in previous similar studies [28,29]. Wet-bulb globe temperatures (WBGT) were continuously measured using a hand-held electronic heat stress monitoring device (Extech, HT30, Heat Stress WBGT Meter, Extech Instruments; Nashua, NH) during the SSGs. All SSGs were performed on a synthetic grass pitch at a similar time (between 8 and 10 a.m.) to avoid the effects of circadian rhythms [30]. The players were familiar with using HR monitors and psychophysiological scales during their daily training routine for at least 3 years. They were also instructed to maintain their usual daily dietary intake and to avoid drinking caffeinated beverages during the study. Two hours before the SSGs, fluid intake (500 mL water) was standardized across participants to minimize the confounding effects of hydration status on thermoregulatory and performance responses [31]. The players were not allowed to drink water during the rest of the SSGs.

### Subjects

An a priori power analysis was conducted using G*Power (version 3.1.9.6, University of Düsseldorf, Germany) for a repeated-measures ANOVA design. Assuming a moderate effect size (f = 0.25), an alpha level of 0.05, and a correlation of 0.5 among repeated measures, a total sample size of 19 participants would be required to achieve 80% statistical power. Repeated-measures designs reduce between-subject variability, thereby increasing sensitivity to detect moderate-to-large main effects even with smaller samples. The selected effect size was based on previous studies investigating psychophysiological responses and locomotor demands during SSGs under different environmental conditions, where moderate-to-large effects are typically observed [13,28]. Therefore, the present study included 16 players, which provides sufficient power to detect moderate-to-large effects. Therefore, sixteen male soccer players (age: 16.5 ± 0.5 years; weight: 59.7 ± 6.9 kg; height: 172 ± 6 cm; training experience: 5.8 ± 0.7 years), competing in a regional amateur league team, played 2-a-side and 4-a-side SSGs under three different environmental heat stress conditions. The players were also familiar with a training workload of >4 training units per week (~100 minutes per session) and had been involved in soccer training and league matches for more than 4 years. The eligibility criteria were as follows: (i) participants were not injured during or in the month before the study; (ii) participants did not suffer from any cardiovascular or pulmonary pathology and did not take any drugs or alcohol during the study; and (iii) participants completed all data collection sessions. Before signing the informed consent form, the players and their guardians were fully informed of the research procedure. All participants provided written informed consent to participate in the study. The research was conducted between August 17, 2022, and August 31, 2022. This study was approved by the Research Ethics Committee (E-33490967-044-146674/18.03.2022) and conducted in accordance with the Declaration of Helsinki.

### Procedures

**Fig 1:**
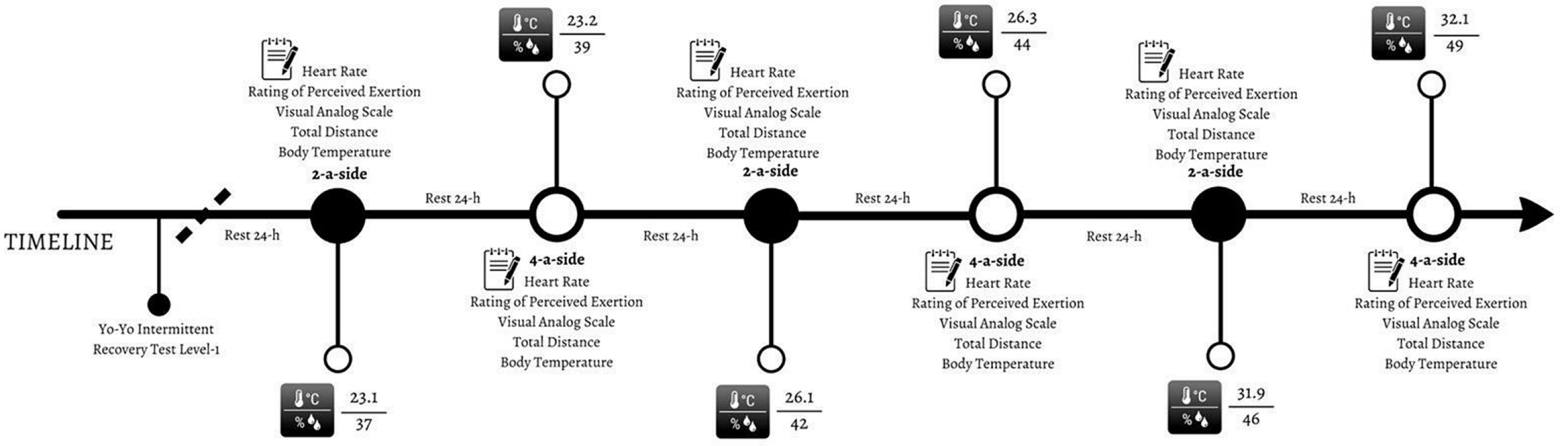
Study design.

In accordance with the research’s purpose, a short-term design (approximately 2 weeks) was used to avoid performance-induced effects during the pre-season. The study design and data on heat-stress conditions, including temperature and relative humidity, are presented in Fig 1.

The environmental conditions, including ambient temperature, relative humidity, radiant heat, and wind speed, were measured using a WBGT monitor that recorded every minute and averaged over the course of the SSGs. The temperatures were also checked and collected using the current information from the Turkish State Meteorological Service. The tympanic temperature (TT) of the players was measured using an infrared tympanic membrane thermometer (ThermoScan 5-IRT6020; Braun, Kronberg, Germany) within 1 minute before the start of the SSGs and within 1 minute after each SSG. The tympanic membrane method (test re-test typical error = 0.22°C, coefficient of variation = 0.61%, assessed in our laboratory) is the safest, easiest, and most invasive method used instead of body core temperature measurement, which is determined using various measurement methods [32,33]. Before each use, the disposable plastic filters of the measuring devices were changed to prevent infection from the players. The differences in TT before and after starting SSGs (post–pre) were calculated for each participant in each SSG.

The HR responses, including average HR (HR_mean_), percentage of HR (%HR_max_), and TDC, were continuously collected and recorded using an HR monitor (Polar M430, Polar Electro Oy, Kempele, Finland) with an H10 chest strap during each SSG. These monitors, which have a global positioning system (GPS), were worn on the left wrist to measure players’ HR responses (1-second interval) [34] and TDC (typically ~1 Hz sampling under sport mode) during all SSGs [35]. A previous study showed that this watch’s GPS delivered accurate and precise results with an average margin of error of 1.74 m on a standard outdoor athletics track (400 m), compared to other sports watches [35]. Each player used the same HR monitor and chest band to reduce inter-device variability, which is important for ensuring the validity and reliability of product performance measurements [36,37]. The highest HR value was recorded as the HR_max_ during the Yo-Yo Intermittent Recovery Test Level-1 (YYIRT-1) [38]. To assess players’ physical fatigue, the rating of perceived exertion (RPE) was determined using Borg’s 6–20 scale within 15 s of the end of each session [39]. The players provided answers individually to reduce the risk of hearing their teammates’ responses about their perceived effort during the SSGs. In addition, an 11-point visual analog scale (VAS) was used to assess players’ perceived environmental heat at the end of each session (0 = no heat; 10 = very hot). In previous studies, these validated and reliable scales have been frequently used to determine perceived psychophysiological responses [40,41]. Following a standardized 10 minutes warm-up section [42] consisting of low-intensity running and dynamic stretching with short-distance passing, all SSGs were performed on a synthetic grass pitch with small goals (1 × 1.5 m) without any special rules. Each team in the SSGs, including two- and four-player teams, was selected by its coach to avoid unbalanced groups. They played with the same teammates and faced the same team members during the study. The 2-a-side (four 2-minute bouts with 2 minutes passive rest) and 4-a-side (four 4-minute bouts with 4 min of passive rest) SSGs were conducted with two different format dimensions with a 100 m^2^ relative pitch size per player: 15 × 27 m for the 2-a-side and 25 × 32 m for the 4-a-side formats. Their coaches provided verbal encouragement to exert maximal effort during SSGs [43].

### Statistical analyses

Data are reported as means ± standard deviation (SD). The normality of the data distribution was assessed using the Shapiro–Wilk test. Sphericity assumptions were evaluated using Mauchly’s test, and when the assumption of sphericity was violated, Greenhouse–Geisser corrected degrees of freedom and p values were reported. Specifically, Greenhouse–Geisser corrections were applied for TDC and TT responses, whereas the sphericity assumption was met for the remaining dependent variables. A two-way repeated-measures analysis of variance (ANOVA) was performed separately for each SSGs format (2-a-side and 4-a-side) to examine the effects of environmental temperature (LEH, MEH, and HEH) and SSGs bout (four bouts), as well as their interaction (temperature × bout) on psychophysiological responses and locomotor demands. When significant main or interaction effects were identified, Bonferroni-adjusted pairwise comparisons were conducted. The effect size was calculated using the partial eta squared ($\overset{\text{2}}{\underset{p}{\eta }}$), interpreted as follows: ≥0.01 indicates a small effect; ≥0.059, a medium effect; and ≥ 0.138, a large effect [44]. Statistical analyses were performed using SPSS software (version 26.0, IBM Corp., Armonk, NY, USA). The level of statistical significance was set at p ≤ 0.05.

## Results

**Fig 2:**
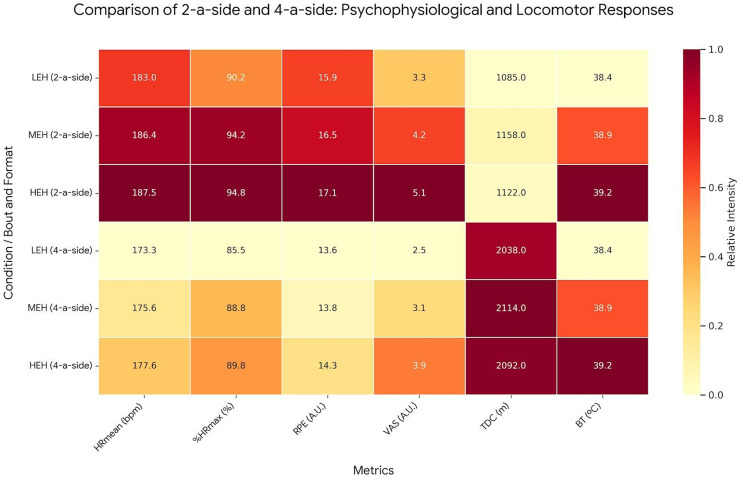
Comparison of 2-a-side and 4-a-side: Psychophysiological and Locomotor Responses.

Fig 2 presents data on psychophysiological responses, locomotor demands, and tympanic temperatures for different SSGs across three heat stress conditions. For the 2-a-side SSGs, a significant main effect of temperature was observed for HR_mean_ (*F*= 9.633; *p* < 0.001; $\overset{\text{2}}{\underset{p}{\eta }}\;$= 0.391), %HR_max_ (*F* = 31.835; *p* < 0.001; $\overset{\text{2}\;}{\underset{p}{\eta }}$= 0.680), RPE (*F* = 13.997; *p* < 0.001; $\overset{\text{2}}{\underset{p\;}{\eta }}$= 0.483) and, VAS (*F* = 48.498; *p* < 0.001; $\overset{\text{2}}{\underset{p}{\eta }}\;$= 0.764) responses. However, no significant main effect of temperature was found for TDC (*F* = 3.389; *p* = 0.071; $\overset{\text{2}}{\underset{p}{\eta }}\;$= 0.184) and TT (*F* = 0.238; *p* = 0.790; $\overset{\text{2}\;}{\underset{p}{\eta }}$= 0.016) responses.

Similarly, in the 4-a-side SSGs, a significant main effect of temperature was found for HR_mean_ (*F* = 7.750; *p* < 0.001; $\overset{\text{2}}{\underset{p}{\eta }}\;$= 0.341), %HR_max_ (*F* = 25.437; *p* < 0.001; $\overset{\text{2}}{\underset{p}{\eta }}\;$= 0.629), RPE (*F* = 12.418; *p* = 0.010; $\overset{\text{2}}{\underset{p}{\eta }}\;$= 0.139), and VAS (*F* = 33.241; *p* < 0.001; $\overset{\text{2}}{\underset{p}{\eta }}\;$= 0.689) responses, whereas no significant effects were observed for TDC (*F* = 2.193; *p* = 0.152; $\overset{\text{2}}{\underset{p}{\eta }}$ = 0.128) and TT (*F* = 1.196; *p* = 0.316; $\overset{\text{2}}{\underset{p}{\eta }}$ = 0.074) responses.

**Fig 3:**
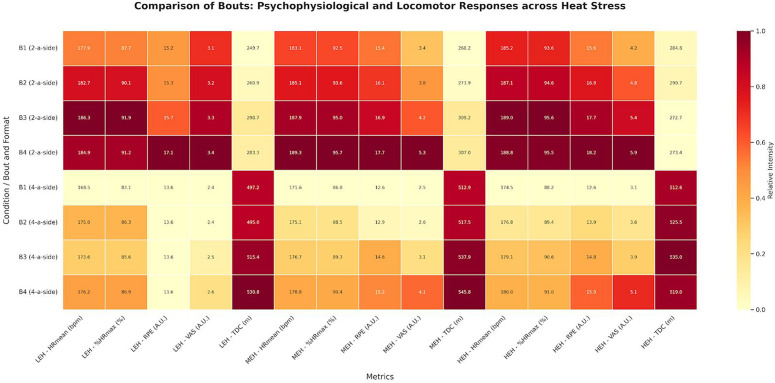
Comparison of bouts: Psychophysiological and Locomotor Responses across Heat Stress.

Regarding interaction effects, a significant temperature × bout interaction was observed in the 2-a-side SSGs for HR_mean_ (*F* = 3.940; *p* = 0.003; $\overset{\text{2}}{\underset{p}{\eta }}$ = 0.149), HR_max_ (*F* = 3.793; *p* = 0.003; $\overset{\text{2}}{\underset{p}{\eta }}\;$= 0.144), RPE (*F* = 2.527; *p* = 0.024; $\overset{\text{2}}{\underset{p}{\eta }}$ = 0.101), VAS (*F* = 5.401; *p* < 0.001; $\overset{\text{2}}{\underset{p}{\eta }}$ = 0.194), and TDC (*F* = 2.892; *p* = 0.017; $\overset{\text{2}}{\underset{p}{\eta }}\;$= 0.114) responses. Similarly, significant temperature × bout interactions were found in the 4-a-side SSGs for HR_mean_ (*F* = 3.432; *p* = 0.006; $\overset{\text{2}}{\underset{p}{\eta }}$ = 0.132), HR_max_ (*F* = 3.321; *p* = 0.008; $\overset{\text{2}}{\underset{p}{\eta }}\;$= 0.129), RPE (*F* = 7.829; *p* < 0.001; $\overset{\text{2}}{\underset{p}{\eta }}$ = 0.258), and VAS (*F* = 7.710; *p* < 0.001; $\overset{\text{2}}{\underset{p}{\eta }}$ = 0.255) responses, whereas no significant interaction effect was observed for TDC (*F* = 0.727; *p* = 0.628; $\overset{\text{2}}{\underset{p}{\eta }}$ = 0.031) responses (Fig 3).

## Discussion

This study contributes to the limited literature examining the effects of environmental heat stress on performance and psychophysiological responses in young male soccer players during SSGs. The research findings revealed that HR responses and TDC were significantly lower in cooler conditions, whereas no significant differences were observed between the two hotter conditions. RPE and VAS responses showed a progressive, significant increase across different heat stress conditions. Additionally, HR, RPE, and VAS progressively increased from the first to the fourth bouts (within the SSG format and heat-stress conditions). However, HR stabilized from the third to the fourth bouts, which could indicate a transition from the parasympathetic to the sympathetic domain of HR modulation [12,45]. This was generalized across different heat-stress conditions and SSG formats. Considering the locomotor demands, TDC showed a progressively increasing tendency across bouts, although this was not evident in hotter conditions.

Increases in TT after SSGs were observed in our study. Comparisons between heat stress conditions for the pre-post difference in TT did not show statistically significant differences. Although values obtained at the end of SSGs in the hotter scenarios are in average greater (39.2°C and 39.2°C in 2-a-side and 4-a-side SSGs formats, respectively), than the cooler (38.4°C and 38.4°C in 2-a-side and 4-a-side SSGs formats, respectively), the difference between starting and ending SSGs session was not significantly different (raises about 1.6°C and 1.8°C across the different temperature conditions). The average TT reported in our study was similar to that reported in a study that measured muscle temperature during matches at 21°C and 43°C [6]. An increase in skin temperature after exercise is expected due to changes in thermal homeostasis induced by alterations in metabolic rate and the consequent increase in internal heat [8]. Thus, blood flow is redirected to the skin to favor heat exchange with the environment, although muscle competition for blood is still required to sustain the effort [46]. For example, in the early stages of exercise, a reduction in skin temperature is expected due to the skin vasoconstriction reflex, which redirects blood flow to active muscles [47]. However, during high-intensity exercises such as SSGs, an increase in skin temperature results from heat dissipation from active muscles to the skin surface [8]. This suggests our study by showing that an increase in skin temperature was consistently observed and did not differ significantly across conditions.

One consequence of exercise stress combined with high temperatures is exercise-induced gastrointestinal syndrome [48]. Our research showed that discomfort increased significantly with incremental heat stress. These findings are consistent with those showing that exertional heat stress exacerbates the primary causal mechanism of exercise-induced gastrointestinal syndrome [49]. This can be specifically justified by the redistribution of blood flow to the working muscles and peripheral circulation, resulting in a reduction in total splanchnic perfusion and possible gastrointestinal ischemia [50,51]. Another hypothesis relates to a reduction in gastrointestinal functional capacity due to suppression of myenteric and submucosal plexus activity [48]. Our research found that environmental conditions played a significant role in modulating psychophysiological responses. In this case, HR responses were significantly smaller in SSGs played at conditions below 23°C, whereas they were similar between conditions at 24 and 33°C. The observation of greater HR responses under hotter conditions is expected because a reduction in stroke volume prompts the body to regulate HR to match cardiac output [5]. These findings are supported by other studies conducted on soccer players. For example, one study [52] tested cool (10°C), mild (22°C), and hot (35°C) conditions during moderate-level exercise, revealing that mild conditions elicited significantly smaller HR responses than the other extreme conditions. These increases in HR responses can also explain the significantly higher RPE scores under hotter conditions, as RPE is strongly related to HR during SSGs [53]. The significant and progressive increase in RPE across environmental conditions can also be explained by the fact that RPE appears to be related not only to the modulation of physiological intensities but also to the rise in BT [54].

During team-sport training and matches, measures of internal load derived from RPE and HR consistently show positive associations with external loads and intensity derived from GPS and accelerometers; however, the strength and uncertainty of these relationships changeable the measure and training mode [55,56]. Interestingly, in contrast to previous research in match scenarios [6,11,57], our findings revealed that TDC was smaller under cooler heat stress conditions than in the remaining heat stress conditions. One justification for players to reduce TDC, particularly the more intense ones, is self-adjustment to cope with heat and physiological stress [58,59]. The context can justify our findings, since in a training scenario and in a small format such as SSGs; players cannot self-pace to the same extent as in a match. Pacing behaviour differs substantially between SSGs and full match-play, with SSGs characterized by aggressive, front-loaded pacing strategies and high variability due to task constraints such as duration knowledge and pitch configuration. In contrast, match scenarios promote a more conservative, regulated pacing profile, in which players distribute effort over time to sustain performance across prolonged durations [60]. Moreover, given that our sample consisted of young athletes, their ability to manage locomotor demands may not be optimal. In fact, a previous study also found that the management of locomotor demands in heat is skill-level-dependent [61].

When analyzing the variation in the main outcomes over repeated bouts of SSGs while interacting with different heat stress conditions, it was found that there was a progressive tendency for increased HR responses over the bouts, with stabilization in the last two. This evidence has been found in control conditions, where the first repetition appears to be less intense than subsequent repetitions, as indicated by HR responses [62,63]. A previous study on this topic argued that the lower HR during the first bout is associated with the time to HR elevation during the bout. In contrast, in the remaining bouts, HR starts at a higher level [63]. However, physiological mechanisms may be a part of this justification. Sympathetic system activity can increase HR responses during repeated bouts, particularly in hotter conditions [45]. Following this tendency, RPE also progressively increases across bouts, with a larger and more significant difference between the first and last bouts, which can be explained not only by physiological mechanisms but also by fatigue perception, which is associated with RPE [64]. Subjective physical fatigue, associated with increased cardiovascular strain, can justify an intensification of the perception of effort [65]. However, such increases in psychophysiological responses do not negatively affect TDC, which progressively increased across repetitions, except in the hotter condition.

One of the primary limitations of the study is the use of infrared tympanic thermometry in outdoor conditions. Although this invasive method is the safest, most practical, and most widely used for measuring body temperature in outdoor conditions, it does not directly reflect core body temperature. It is sensitive to environmental factors, including ambient temperature, wind speed, and airflow. These factors may reduce the method’s sensitivity to detect subtle thermoregulatory changes during exercise in hot conditions. Therefore, the changes in TT observed in this study should be evaluated with the understanding that they may partly reflect methodological limitations. The other limitation is that players in SSGs exhibit highly variable behaviour, which justifies some variability in outcomes. While the Polar M430 includes a 50 Hz triaxial accelerometer, this sampling rate applies specifically to the accelerometer and should not be interpreted as equivalent to GPS sampling frequency. Furthermore, wrist-worn devices both overestimate and underestimate, depending on movement-related metrics and intensity. Therefore, TDC should be interpreted as an estimate of external load trends rather than as an exact positional-tracking measure. Additionally, heat stress conditions were applied in a fixed sequence due to environmental constraints. To minimize potential sequence effects and heat stress-related physical fatigue in players, a short-term research design was employed. Despite these limitations, this is a pioneering study on the effect of heat stress on SSGs, which are typically used by coaches in training scenarios. Possible implications should be extracted for the practice, namely, considering that the hotter scenarios played an essential role in the perception of effort and VAS, as well as in the rise of HR, it can be a strategy to adjust the period of training or even use cooler strategies to avoid increasing the exposure of players to uncomfortable scenarios, which may also compromise performance.

### Conclusions

Heat stress induced by hotter conditions resulted in significant increases in HR, RPE, and VAS responses among soccer players during SSGs in different formats. The interactions between environmental heat stress conditions and the number of bouts per format revealed that HR and RPE tended to increase across repetitions, while TDC showed bout-related variation without significant overall differences between heat conditions. Coaches and sports scientists working in soccer should be aware that performing SSGs in a hot environment affects internal responses independently of the game format.
